# An Efficient Next Hop Selection Algorithm for Multi-Hop Body Area Networks

**DOI:** 10.1371/journal.pone.0146464

**Published:** 2016-01-15

**Authors:** Vahid Ayatollahitafti, Md Asri Ngadi, Johan bin Mohamad Sharif, Mohammed Abdullahi

**Affiliations:** 1 Department of Computer Science, Taft Branch, Islamic Azad University, Taft, Yazd, Iran; 2 Department of Computer Science, Universiti Teknologi Malaysia, 81310 Johor Bahru, Malaysia; 3 Department of Mathematics, Ahmadu Bello University Zaria, Zaria, Nigeria; Banner Alzheimer’s Institute, UNITED STATES

## Abstract

Body Area Networks (BANs) consist of various sensors which gather patient’s vital signs and deliver them to doctors. One of the most significant challenges faced, is the design of an energy-efficient next hop selection algorithm to satisfy Quality of Service (QoS) requirements for different healthcare applications. In this paper, a novel efficient next hop selection algorithm is proposed in multi-hop BANs. This algorithm uses the minimum hop count and a link cost function jointly in each node to choose the best next hop node. The link cost function includes the residual energy, free buffer size, and the link reliability of the neighboring nodes, which is used to balance the energy consumption and to satisfy QoS requirements in terms of end to end delay and reliability. Extensive simulation experiments were performed to evaluate the efficiency of the proposed algorithm using the NS-2 simulator. Simulation results show that our proposed algorithm provides significant improvement in terms of energy consumption, number of packets forwarded, end to end delay and packet delivery ratio compared to the existing routing protocol.

## Introduction

**Fig 1:**
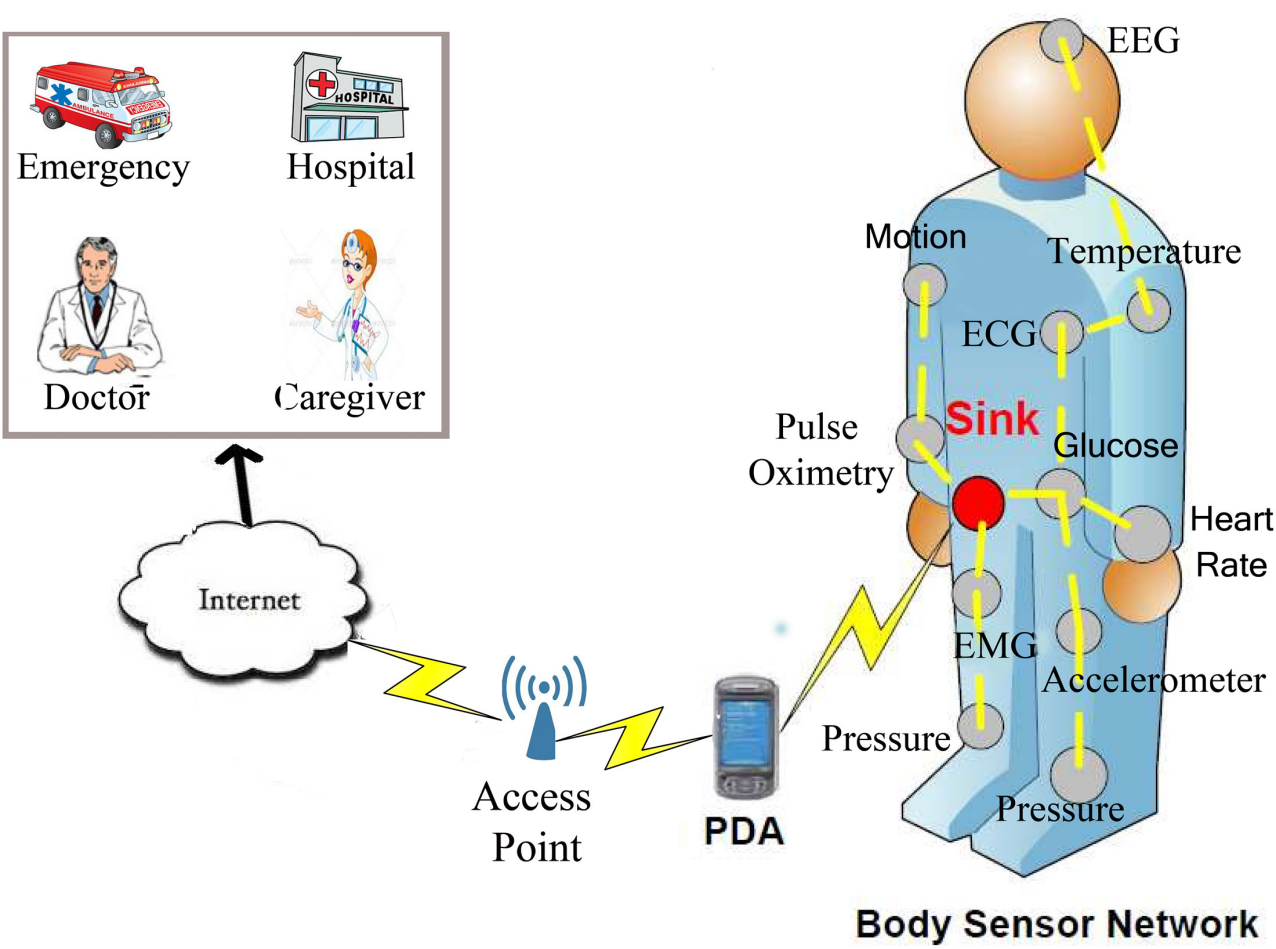
An example of Body Sensor Network.

Due to the growth in healthcare technology and rise in its costs, recognizable attention has been given to the human body with miniaturized, low power and intelligent sensors that can be implanted in or worn on the body. A Body Sensor Network (BSN) or Body Area Network (BAN) is a radio frequency (RF)-based wireless technology which enables monitoring of the patients, whereby physicians or doctors receive information from those patients without disturbing their day to day life (see Fig 1). BAN communications architecture is divided into three components: intra-BAN, inter-BAN, and beyond-BAN [1–3]. Intra-BAN communication controls and manages wearable or implanted sensors. In this tier, the patient’s vital signs are collected and transmitted to the sink. In inter-BAN communication, collected information from the body is forwarded to a gateway. Communications between the gateway and the doctors are related to the beyond-BAN tier. In large-scale networks of BANs specially in the inter-BAN and the beyond-BAN communications, cloud computing infrastructure can facilitate network management [4]. Cloud computing infrastructure is able to deal with the most important challenges posed by the management of large-scale networks of BANs. Integration of BAN and cloud computing technology can provide flexible data processing and management to perform both online and offline analysis of body sensor data streams [5, 6]. Cloud computing technology promotes different BANs applications to integrate more smart stations and provide more convenience and entertainment for patients [7].

Due to the short battery life span of the sensors on the body, optimal energy consumption is a crucial problem in BANs [8]. If a sensor on the body runs out of battery while it is sending vital signs or physiological signals, it would present unsatisfactory [9]. Also in applications such as remote surgery, life-critical or medical disaster applications that require real-time monitoring of the patients, sending reliable information, regularly and on time is very important [10–12]. These metrics (i.e. delay and reliability) are referred to as QoS requirements [13]. QoS refers to the capability to provide assurance that the service requirements of applications can be satisfied. QoS parameters such as delay and reliability can also be used to measure the system performance and to control that the QoS is actually been provided by it [14, 15]. Consider a disastrous situation in which the data related to a patient in critical condition does not reach its destination on time or is corrupted by any means, and ultimately results in its demise.

Resource limitations, communication range, and path loss are a number of BAN characteristics [11]. Due to the short communication range and high path loss in BANs, direct communication between the nodes and the sink node requires additional energy consumption [16]. Thus, sensor nodes in a BAN should transfer data to the sink node with the multi-hop communications. Traditional end-to-end path discovery based routing protocols are not suitable for BANs because of the resource limitations of these networks [17]. Hence, BANs should implement hop-by-hop routing to consume energy more efficiently. According to the above mentioned characteristics of BANs, energy-efficient QoS aware routing is a big challenge in multi-hop BANs.

Existing energy-efficient QoS aware routing protocols in WSNs cannot be applied directly to BAN communications with respect to the BAN characteristics. On the other hand, current hop-by-hop routing protocols in BANs do not efficiently select next hop node in order to satisfy energy and QoS requirements.

We propose an Energy-efficient Next hop Selection Algorithm for multi-hop BAN, ENSA-BAN, in which QoS requirements such as delay and reliability are also considered. The proposed protocol utilizes hop counts and link cost of the neighboring nodes to select the best next hop node for packets forwarding. This protocol uses residual energy, available queue size, and link reliability of a neighboring node to calculate its link cost. Each node selects an appropriate node among its neighboring nodes as the next hop node which has the minimum hop counts to the sink and the maximum link cost. Therefore, the routing algorithm considers not only the QoS requirements, but also the energy consumption of the nodes to improve the network performance. We evaluated our routing algorithm with several experiments in NS-2 simulator. The results indicated that the proposed algorithm outperforms the existing algorithms in terms of average energy consumption, end-to-end delay, packet delivery ratio and number of packets forwarded.

## Related Work

There are many challenging requirements such as security, routing, and QoS that need to be considered in BANs. Since health data of the patients should be private, the security of data transmission within a BAN is a critical issue. A series of research work on deploying secure WBANs applications have been proposed [18–24]. Routing strategies in BANs can be categorized in five groups, namely: thermal-aware, delay tolerant, cluster based, cross layer, and QoS aware routing [25, 26]. QoS aware routing is one of the major challenges in BANs because of the limited resources of the sensors and QoS requirements in healthcare applications [1, 11, 27].

RL-QRP [28], a reinforcement learning based routing protocol, is a QoS-aware routing in biomedical sensor networks. In this protocol, sensor nodes find the optimal path to the sink, using a Q-learning algorithm with regard to the QoS requirements. A geographical and QoS-based routing protocol known as LOCALMOR, was proposed in [29] for biomedical sensor networks. In LOCALMOR, data packets are classified into several categories based on their QoS requirements. The protocol takes into account reliability, residual energy, and latency of the sensor nodes as the QoS metrics. This mechanism has a high overhead leads to additional energy consumption. RTRE [30], a novel framework of real-time data report and task execution, was proposed to collect data through coordination among sensors and mobile actuators. RTRE provides good performance considering delay, energy efficiency and reliability using characteristics of sensors and actuators.

DMQoS (Data-centric multi-objective QoS-aware routing protocol) [17] chooses the next hop node using a multi-objective lexicographic optimization (LO) approach based on the residual energy and the distance to the destination. Data packets are classified into critical packets (CP), ordinary packets (OP), delay-driven packets (DP) and reliability-driven packets (RP). According to the QoS requirements of each type of the data packets, they are forwarded to their next hop nodes.

EPR (Energy efficient peering routing protocol) [31] is a mechanism for peer routing in indoor hospital environments. This routing protocol calculates the communication cost for each neighboring node of a node. Routing table is constructed using this communication cost. QPRR (QoS-aware peering routing protocol for reliability-sensitive data) [32] is another QoS-aware routing protocol in hospital body area networks. QPRR calculates reliability of all paths to the destination and selects a path regarding to the QoS requirements. QPRD (QoS-aware peering routing protocol for delay-sensitive data) [33] handles real-time and non real-time information in hospital body area networks. In this protocol, the end-to-end delay of each path is calculated and the best path is selected for delay-sensitive packets.

Many QoS-aware routing protocols were proposed in WSNs [34]. EQSR (Energy efficient QoS aware routing) protocol [35] is a QoS aware multipath routing protocol which uses different paths according to the QoS requirements of the packets. This protocol classifies traffic to real-time and non-real-time. Every node calculates the cost of its neighboring nodes using a link cost function and selects the next hop node based on the maximum cost. EQSR finds node-disjoint paths from the source node to the sink for different types of the traffic.

ECMP (Energy-constrained multi-path routing) [36] is a QoS-aware routing protocol in which energy consumption is optimized by selecting a path with minimum hop counts in WSNs. MQoSR (A multi-objective QoS routing protocol) [37] is a QoS-aware routing protocol which was proposed in WSNs. MQoSR routing is based on the QoS requirements and geographical information of the nodes. Delay, reliability and energy are QoS requirements related to different applications which are taken into account in this protocol. A few routing protocols which were proposed in intra-BAN communications [38–41] do not encompass QoS requirements.

Mentioned protocols except DMQoS and EPR are end-to-end path discovery-based routing, which requires additional energy consumption. However, DMQoS and EPR protocols do not consider the link reliability between the nodes for ordinary data packets whereby the number of packets retransmitted increases, leads to the higher energy consumption. Furthermore, finding geographical information of the nodes in DMQoS requires specific hardware components, causing extra energy consumption. Therefore, QoS aware and energy-efficient routing in the multi-hop BANs is a challenge which needs to be taken into account.

## Materials and Methods

In this section, the details of the network model and assumptions, the initialization phase, the link reliability, the energy model, the link cost function and routing algorithm of the proposed protocol are provided.

### Network model and Assumptions

A body area network with a single sink and a couple of source nodes is considered in intra-BAN communications. The routing protocol is based on hop-by-hop mechanism. Each source node generates data packets and send them to the next hop node. The intermediate nodes relay and forward the packets to the next hop node until it reaches the sink. All nodes are stationary and have the same transmission range. Additionally, all nodes at any given time are able to calculate their residual energy and free buffer size. Also each node can compute its link reliability between itself and its neighboring nodes. The basic notations used in this study is summarized as follows:

*N*, the number of sensors;
*S* = {*s*_1_, *s*_2_, …, *s*_*N*_}, the set of sensors;
*s*_*i*_, the *i*th sensor, 1 ≤ *i* ≤ *N*;
*N*_*i*_ = {*n*_1_, *n*_2_, …, *n*_*k*_}, the neighboring set of node *s*_*i*_, 1 ≤ *k* ≤ *N*;
*E*_*res*, *i*_, the residual energy of the node *s*_*i*_;
*Q*_*empty*, *i*_, the free queue size of the node *s*_*i*_;
*HOP*_*min*, *i*_, the minimum hop count to the sink from the node *s*_*i*_;
*LinkR*_*ij*_, the link reliability between the nodes *s*_*i*_ and *s*_*j*_;
*Cost*_*ij*_, the link cost between the nodes *s*_*i*_ and *s*_*j*_;
*SN*_*i*_, the selected set of *N*_*i*_;
*NH*_*i*_, the next hop node of *N*_*i*_.

### Initialization phase

**Fig 2:**
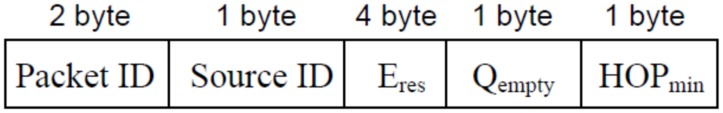
Hello message structure.

Each sensor node generates HELLO packets and broadcasts them periodically to its neighboring nodes. The most important fields of a HELLO packet are shown in Fig 2. *PacketID* is the sequence number of the Hello packet. This number prevents receiving a duplicate packet in the neighboring nodes. *SourceID* is the identifier of the source node of the packet. Residual energy (*E*_*res*_) is the residual energy of the node, which is computed using Eq 3. Free queue size (*Q*_*empty*_) field is the available size of the queue in the node. Minimum number of hops to the sink (*HOP*_*min*_) is computed and is put in the last field. *HOP*_*min*_ is defined by Eq 1 as follows:

$$HOP_{min,i}=\left\{minimum\left(HOP_{j}\right)|j\in N_{i}\right\}+1$$ (1)

where *HOP*_*j*_ is the minimum hop counts of node *s*_*j*_ to the sink node. In this Eq 1 is added to the minimum hop counts of the neighboring nodes because the hop count of node *s*_*i*_ to its neighboring nodes is 1.

**Fig 3:**
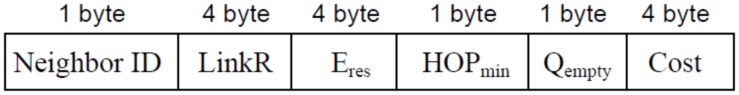
Neighbor table structure.

Upon reception of a HELLO packet in a node, that node adds a new entry in its neighbor table. If an entry exists in the neighbor table for that node, the information of that node is updated according to the HELLO packet fields. Neighbor table structure is illustrated in Fig 3. *NeighborID* is identifier of the neighboring node. *LinkR* is the link reliability between two nodes, which is obtained from Eq 2. *E*_*res*_, *HOP*_*min*_, and *Q*_*empty*_ fields are extracted from Hello packets. *Cost* field is the link cost of the neighboring nodes, which is obtained from Eq 7.

### Link reliability

Link reliability between two nodes affects the QoS requirements and the energy consumption because the low link reliability causes the high retransmitted packets whereby energy consumption is increased. The link reliability between two nodes (*LinkR*_*ij*_) is calculated using the exponentially weighted moving average (EWAD) by Eq 2.

$${LinkR}_{ij}=\left(1-\gamma \right){LinkR}_{ij}+\gamma \left(\frac{{Tx}_{succ,ij}}{{Tx}_{total,ij}}\right)$$ (2)

where *Tx*_*succ*, *ij*_ is the number of packets successfully transmitted through the link between node *s*_*i*_ and node *s*_*j*_, *Tx*_*total*, *ij*_ is the total number of transmission and retransmission attempts for all packets and *γ* is the average weighting factor. The value of *γ* is set to 0.4 in our simulation.

### Energy model

To balance energy consumption between sensor nodes, the residual energy of the nodes is taken into account. Residual energy of node *s*_*i*_ (*E*_*res*, *i*_) is given by

$$E_{res,i}=E_{init,i}-E_{con,i}$$ (3)

where *E*_*con*, *i*_ is the energy consumed inside the node *s*_*i*_. To calculate the energy consumption in a node, total amount of transmission and reception energy in a node is computed [42]. *E*_*con*, *i*_ is given by Eq 4.

$$E_{con,i}=a_{i}\times E_{tx}+b_{i}\times E_{rx}$$ (4)

where *a*_*i*_ and *b*_*i*_ are the number of bits transmitted and received in the node *s*_*i*_. *E*_*tx*_ and *E*_*rx*_ are defined by

$$E_{tx}=E_{tx_{elec}}+E_{amp}\times d^{2}$$ (5)

$$E_{rx}=E_{rx_{elec}}$$ (6)

where *E*_*tx*_*elec*__ and *E*_*rx*_*elec*__ are the energy which the radio needs for the transmitter and the receiver respectively, *E*_*amp*_ is the energy for the transmit amplifier, and *d* is distance between nodes *s*_*i*_ and *s*_*j*_.

### Link cost function

The link cost function is utilized in each node to find the next hop node. This function includes residual energy of node *s*_*j*_, free queue size of node *s*_*j*_ and link reliability between nodes *s*_*i*_ and *s*_*j*_. *Cost*_*ij*_ is defined as follows:

$$Cost_{ij}=C_{E}\times \frac{E_{res,j}}{E_{init,j}}+C_{Q}\times \frac{Q_{empty,j}}{Q_{total,j}}+C_{L}\times LinkR_{ij}$$ (7)

where *E*_*res*, *j*_, *E*_*init*, *j*_, *Q*_*empty*, *j*_, and *Q*_*total*, *j*_ are residual energy, initial energy, available queue size, and maximum queue size of node *s*_*j*_ respectively. *LinkR*_*ij*_ is the link relibility between two nodes *s*_*i*_ and *s*_*j*_. Furthermore, *C*_*E*_, *C*_*Q*_ and *C*_*L*_ are three constant coefficients.

In the link cost function, three factors are considered to satisfy QoS requirements. Residual energy of the nodes is used to balance the energy between the nodes. Queue size of the nodes is considered in the cost function because the queuing delay has a significant contribution in the end-to-end delay. Also link reliability of the nodes increases the reliability of the network.

The objective link cost function for node *s*_*i*_ is defined by Eq 8.

$$maximize\;\left({Cost}_{ij}\right),\;\forall \;j\in SN_{i}\;$$ (8)

where *SN*_*i*_ is obtained from the Eq 9.

$$SN_{i}=\left\{j|\forall j\in N_{i},HOP_{min,j}=HOP_{min,i}-1\right\}$$ (9)

where *N*_*i*_ is the neighboring list of node *s*_*i*_ and *HOP*_*min*, *j*_ is the minimum hop counts of node *s*_*j*_ to the sink node.

### Routing algorithm

After a node receives a HELLO packet from its neighboring node, the node updates its neighbor table as mentioned before. The node selects new next hop node periodically. The next hop selection algorithm is presented in Algorithm 1. In the first loop, link cost of all neighbors of the node *s*_*i*_ (*Cost*_*ij*_) is calculated using Eq 7. In the second loop, the neighboring nodes which the hop counts to the sink through them is the minimum hop count, are added into the set of the selected neighboring nodes (*SN*_*i*_). *Hop*_*min*, *i*_ is obtained by HELLO packets. After that, *SN*_*i*_ is sorted in descending order of *Cost*_*ij*_. Finally, the first element of the set is selected as the new next hop node. This mechanism satisfies QoS requirements since it uses the link cost of neighboring nodes (residual energy, queue length and link reliability) and the minimum hop count to the sink.

Algorithm 1 *The pseudo code for next hop node selection*

**INPUT:** *N*_*i*_, *Hop*_*min*, *i*_, *SN*_*i*_

**OUTPUT:** *NH*_*i*_

1:  **for** (All nodes in list *N*_*i*_) **do**

2:   Compute *Cost*_*ij*_, *j* ∈ *N*_*i*_

3:  **end for**

4:  *j* = first element of *N*_*i*_

5:  **while** (Not end of list *N*_*i*_) **do**

6:   **if** (*HOP*_*min*, *j*_+1 == *HOP*_*min*, *i*_) **then**

7:    add *j* to *SN*_*i*_

8:   **end if**

9:   *j* = next element of *N*_*i*_

10:  **end while**

11:  Sort *SN*_*i*_ (in descending order of *Cost*_*ij*_)

12:  *NH*_*i*_ = First element of the list *SN*_*i*_

**Fig 4:**
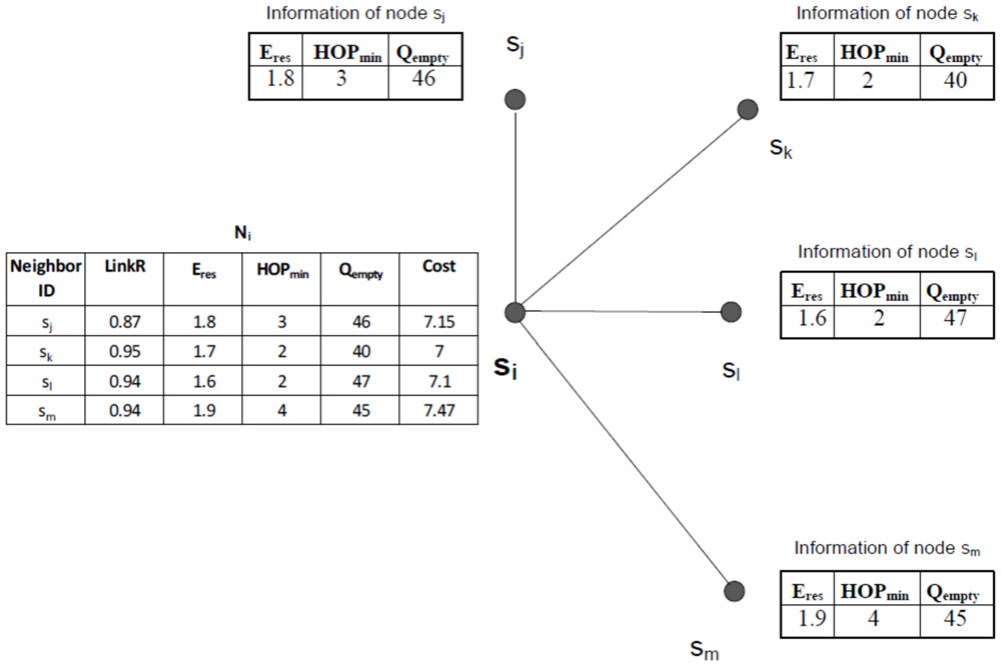
An example of next node selection.

An example is shown in Fig 4 to illustrate the next hop node selection algorithm. Assume that the node *s*_*i*_ needs to select a new next hop node. When this node receives information from its neighboring nodes namely *s*_*j*_, *s*_*k*_, *s*_*l*_, and *s*_*m*_ by the HELLO packets, node *s*_*i*_ calculates the link cost of its neighboring nodes. The link reliability between node *s*_*i*_ and its neighboring nodes is computed inside node *s*_*i*_ using Eq 2. Assume that *HOP*_*min*, *i*_ is equal to 3. According to the above algorithm, nodes *s*_*k*_ and *s*_*l*_ are added to *SN*_*i*_. After sorting *SN*_*i*_ in descending order of the cost, the first element of *SN*_*i*_ which is node *s*_*l*_ is selected as the new next hop node.

## Performance Evaluation

### Setting and Configuration

**Fig 5:**
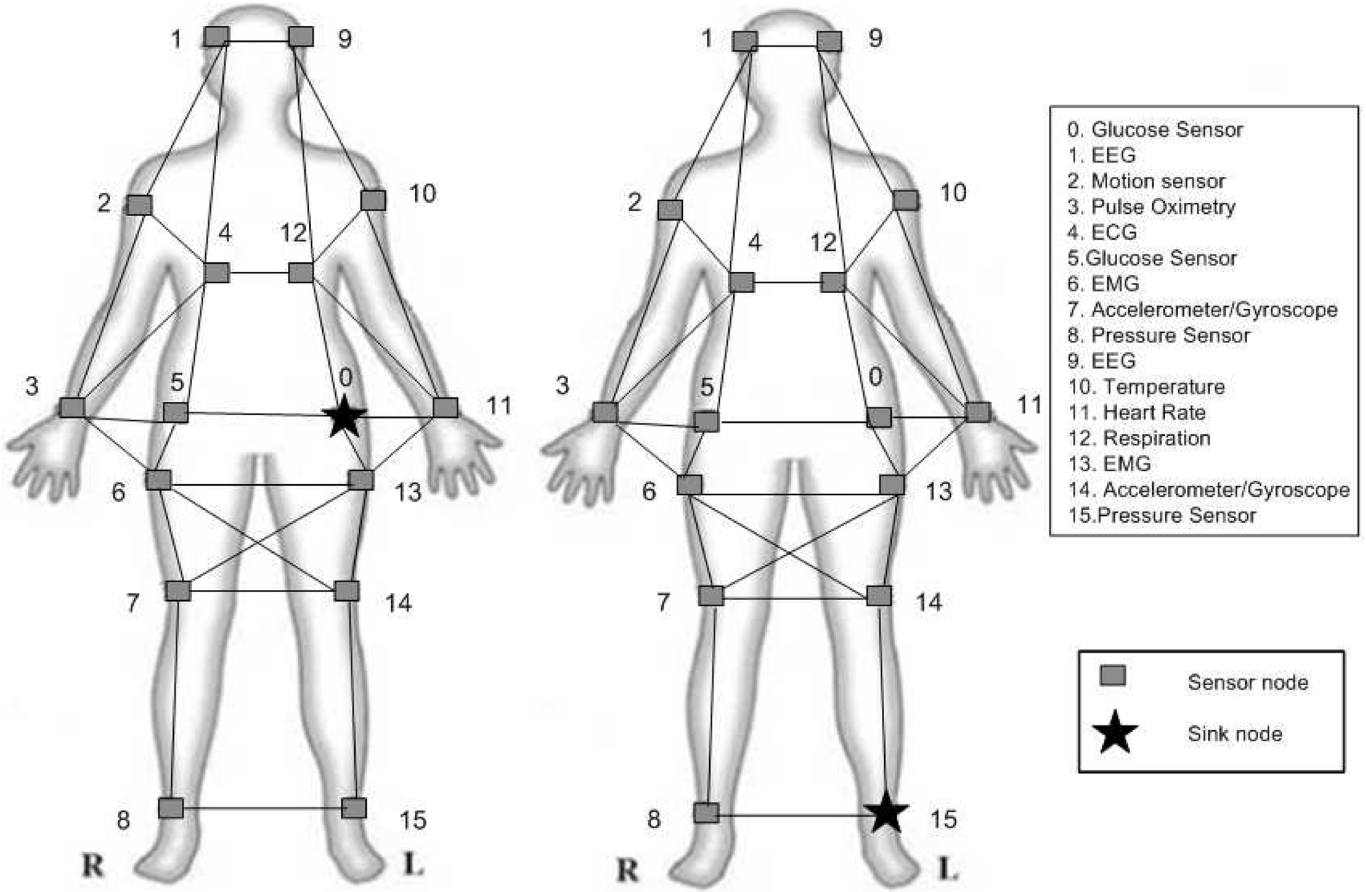
Network model consisting of 15 sensor nodes and one sink node located at (a) waist (b) ankle.

The performance of the proposed protocol is studied and compared with the DMQoS [17] using simulator NS-2 [43]. Two main experiments were performed to assess the network. In each experiment, 16 sensors were deployed in 2 × 2*m*^2^ body area with mesh topology as shown in Fig 5.

In the first experiment, the sink node was located at the patient’s waist (Fig 5(a)) and in the second experiment; the sink node was located at the patient’s ankle (Fig 5(b)). Each simulation experiment was run 50 times over. In the physical layer, MicaZ motes are used which are based on CC2420 radio chip. Propagation model is free space and path loss of the propagation model is set to 2 dB. In the link layer, maximum transmission rate is 250 Kbps and frequency band is 2.4 GHZ. The sensors are communicating each other with a coverage range of 70 cm. The IEEE standard 802.15.4 is used for MAC layer and CSMA/CA mechanism is used for collision avoidance. Other simulation parameters for two experiments are shown in Table 1.

**Table 1:** Simulation Parameters.

*Parameter*	*Value*
Traffic type	CBR
Mobility	None
Queue length	50
Initial node energy	2 Joules
Packet size	32 Bytes
Simulation time	200 sec
*C*_*E*_, *C*_*Q*_, *C*_*L*_	3, 2, 3
Transmission power	0.3 mW
MAC protocol	IEEE 802.15.4

### Performance metrics

In order to evaluate the performance of the proposed routing protocol, following metrics were used in the simulation.

#### Energy consumption

Since sensor nodes in/on the human body have limited energy, energy consumption is one of the important factors which need to be taken into account in BANs. If a sensor stops working because of the low power of the battery, vital signs of the patient will not be accessible, resulting in an undesirable situations.

#### Packets forwarded

This metric shows the number of packets that are forwarded by the intermediate nodes. The more the intermediate nodes, the more the energy consumption and the more the delay in the network.

#### End-to-end delay

This metric is the total latency experienced by a packet from the source node to the sink. Because some applications such as disaster, emergency and remote surgery applications are time-critical, delay is an important factor and one of the QoS requirements which should be considered in BANs. End-to-end delay is the sum of the queuing delay, the processing delay, the transmission delay, and the propagation delay.

#### Packet Delivery Ratio (PDR)

This metric is the ratio of the number of successfully delivered data packets to the sink over the total number of packets generated by all sources. High percentage of the packet delivery ratio increases network reliability and satisfies the QoS requirements better. In the healthcare applications, high percentage of this metric is very important to deliver reliable vital signs of the patients to the doctors.

### Experimental results

In this section, two experiments are presented to verify the performance of the proposed routing algorithm. In the first experiment, the sink node is located on the patient’s waist and the amount of energy consumption and QoS metrics are obtained and discussed. In the second experiment, the sink node is located on the patient’s ankle and also the amount of energy consumption and QoS metrics are obtained and discussed. After that, a comparison between these two experiments are provided and discussed.

#### Experiment1

**Fig 6:**
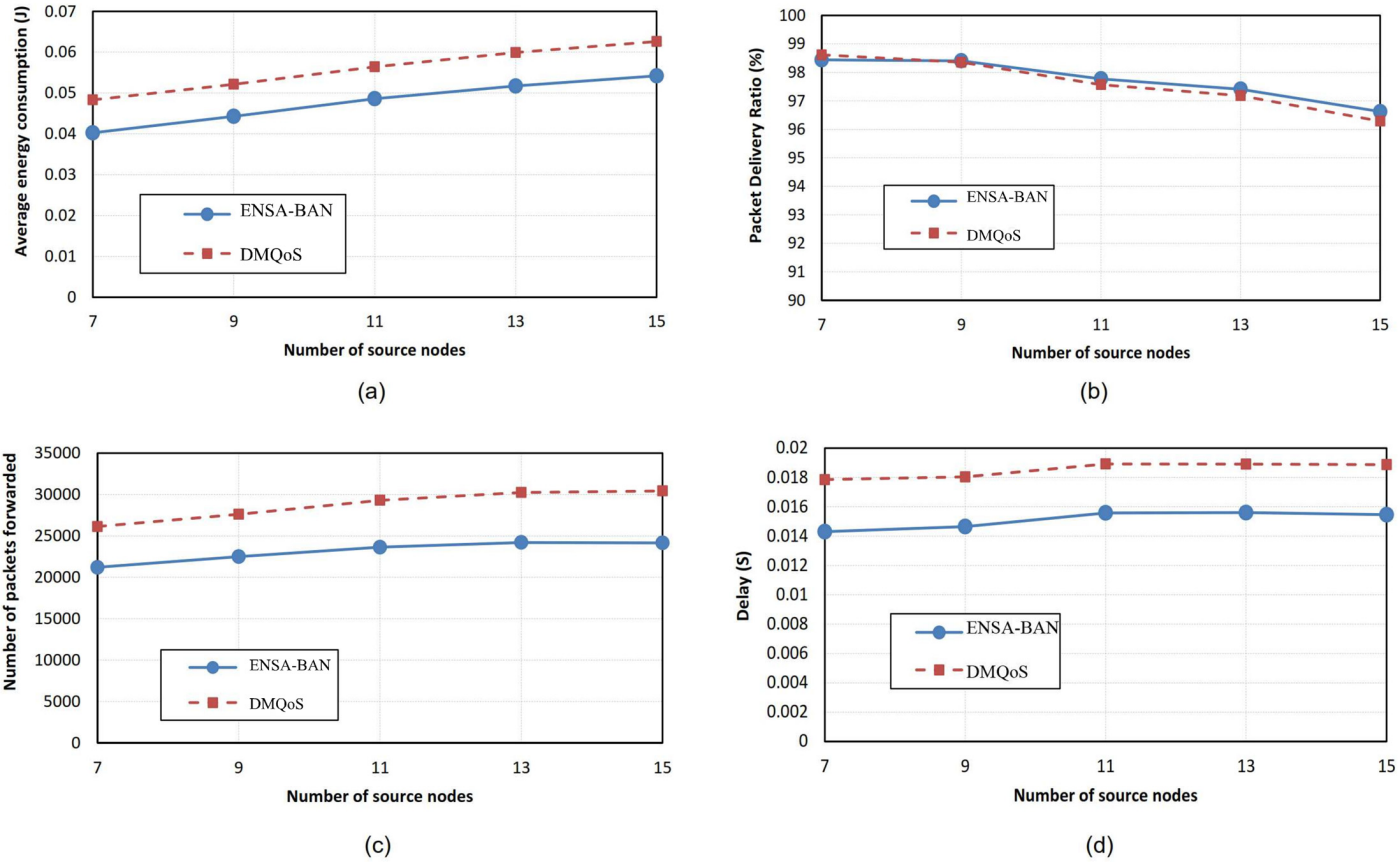
Performance comparisons for varying number of source nodes (sink located at the waist).

In this experiment, the sink is located at the patient’s waist. The data in S1 Dataset was used for the experiment. Average energy consumption of the nodes versus the number of source nodes is shown in Fig 6(a). ENSA-BAN has lower energy consumption (approximately 15%) compared to the DMQoS protocol. This is because in ENSA-BAN when a node decides to select the next hop node, that node considers the residual energy and minimum hop count of its neighboring nodes. The residual energy in the link cost function balances energy consumption between the nodes in the network. The number of hop counts to the sink has an indirect effect on energy consumption. The lower hop count decreases the number of packets forwarded between the nodes, thereby consuming lower energy.

Fig 6(b) shows the packet delivery ratio under different number of source nodes. As it can be observed from the graph, the packet delivery ratio of ENSA-BAN is slightly larger than the another. In the link cost function of ENSA-BAN, considering the link reliability of the neighboring nodes leads to selection of a neighboring node with the better link quality. The higher the link quality, the lower the packet loss, leading to the higher the packet delivery ratio. Another parameter which is considered in ENSA-BAN is the number of packets forwarded by intermediate nodes. If the number of packets forwarded decreases, the energy consumption, required for forwarding the packets, also decreases. As it can be seen from Fig 6(c), this metric in ENSA-BAN is lower than the DMQoS protocol (about 25%). In ENSA-BAN, a node with minimum number of hops to the sink is selected as the next hop whereby the packet forwarded is reduced. Fig 6(d) shows the average end to end delay of ENSA-BAN and DMQoS protocols for varying number of source nodes. As it can be seen from the graph, the average end to end delay for ENSA-BAN is about 20% lower than the DMQoS protocol. This is because, unlike the DMQoS protocol, the ENSA-BAN protocol takes into account the queue size of the neighboring nodes in the link cost function to select the next hop node. As mentioned before, queuing delay has a significant contribution in end-to-end delay. Furthermore, selecting next hop node with minimum hop counts to the sink node can reduce end to end delay.

#### Experiment2

**Fig 7:**
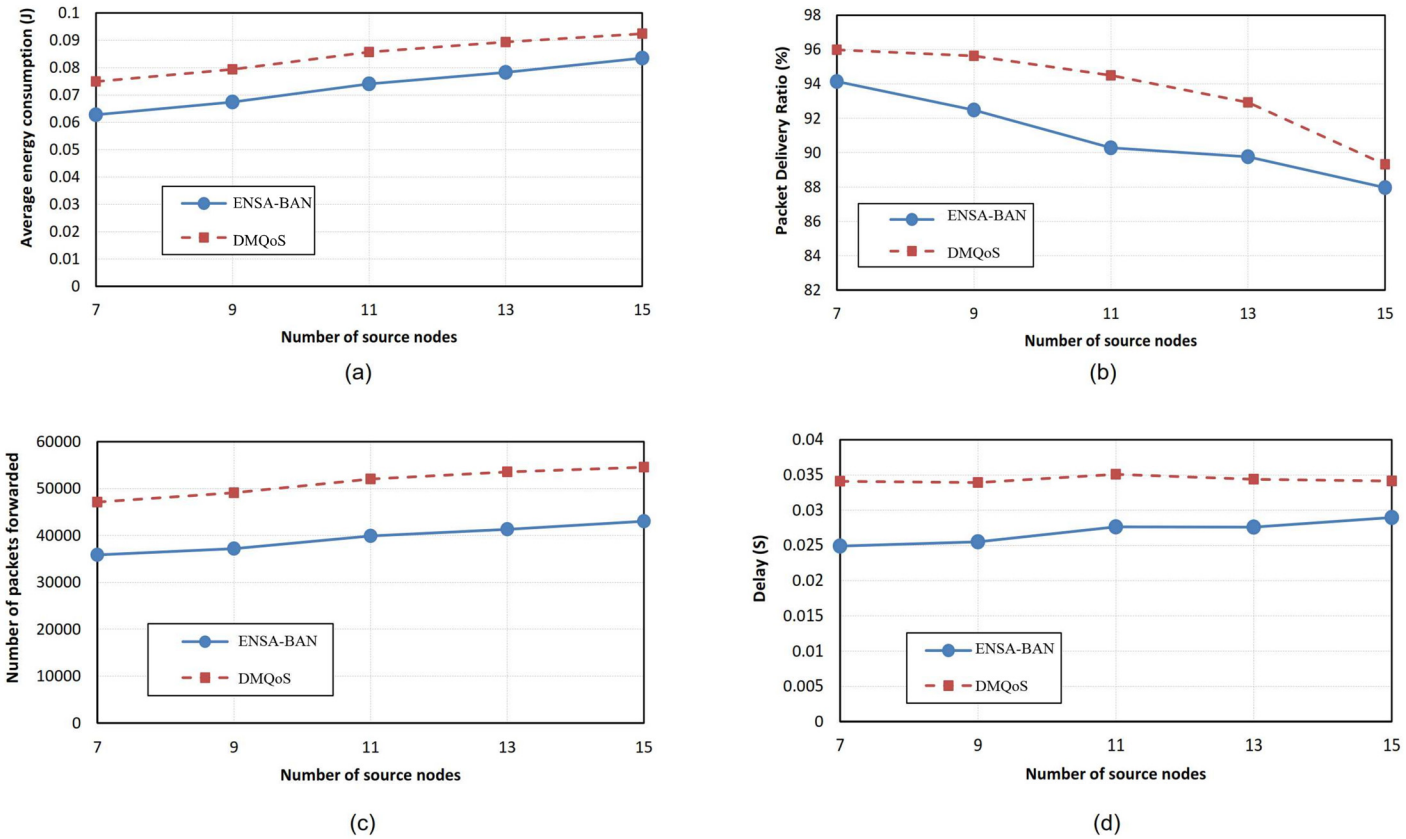
Performance comparisons for varying number of source nodes (sink located at the ankle).

In this experiment, the sink is located at the patient’s ankle. The data in S2 Dataset was used for the experiment. Average energy consumption of the nodes when the sink node is located at the patient’s ankle is shown in Fig 7(a). The ENSA-BAN protocol has lower energy consumption (approximately 10%) compared to the DMQoS protocol. As mentioned before, this is because the ENSA-BAN selects the next hop node considering both residual energy and minimum hop counts. Fig 7(b) shows the packet delivery ratio of ENSA-BAN and DMQoS. The graph shows the packet delivery ratio of ENSA-BAN is lower than another protocol. This is because when the sink node is located at the patient’s ankle, the hop counts of the nodes are increased and the path with minimum hop counts is not necessarily the path with high reliability. This is one drawback of the proposed protocol, when the sink node is located at the patient’s ankle. Fig 7(c) shows the number of packets forwarded by the intermediate nodes. This metric in the ENSA-BAN protocol is 27% lower than the DMQoS protocol, because in the proposed algorithm, a node with the minimum hop counts is selected as a next hop. Fig 7(d) shows the average end to end delay of the ENSA-BAN and DMQoS protocols. As it can be observed from the graph, the average end to end delay for ENSA-BAN is about 18% to 33% (according to the number of source nodes) lower than the DMQoS protocol. As mentioned before, two factors have the effect on delay in the proposed protocol. First factor is the free queue size of the neighboring nodes and second one is the minimum hop count which reduces end to end delay.

A comparison between the first and the second experiments are shown in Table 2. The table shows the average energy consumption, packets forwarded, delay, and packet delivery ratio of ENSA-BAN according to the location of the sink node. When the sink node is located at the patient’s ankle, the increase in the number of forwarded packets is 70% because the average hop counts in the experiment 2 is higher than that of in experiment 1. Due to the higher number of forwarded packets in the experiment 2, energy consumption and delay are increased and packet delivery ratio is decreased. It is noteworthy that higher hop counts cause more retransmission attempts for packets in the nodes, leading to higher packet drops.

**Table 2:** Simulation results with changing the sink location.

*Protocol*	*Avg. Energy consumption (J)*	*Avg. packets forwarded*	*Avg. Delay (S)*	*Avg. PDR (%)*
ENSA-BAN (Sink at waist)	0.04783	23134	0.01508	97.77
ENSA-BAN (Sink at ankle)	0.07320	39445	0.02692	91.13

Therefore, when the sink is located at the patient’s waist, the proposed protocol has a better performance in terms of energy consumption and QoS requirements (i.e. delay and reliability) than the ankle.

## Conclusion

The paper has presented an efficient next hop selection algorithm for multi-hop BANs. The proposed algorithm selects the best next hop for each node based on a link cost function and hop counts to the sink of the neighboring nodes. The link cost function takes into account QoS requirements and uses the residual energy, free queue size, and the link reliability of the neighboring nodes. We have evaluated the performance of ENSA-BAN with different network scenarios using NS-2. The results indicate that, the proposed protocol achieves lower energy consumption, lower forwarded packets, lower end to end delay, and higher packet delivery ratio than the DMQoS protocol. Furthermore, results indicate that when the sink node is located on the patient’s waist in the proposed protocol, energy and QoS parameters have an improvement comparing the patient’s ankle location. Ongoing work on this area includes designing a QoS-aware routing protocol considering the body movement and packet prioritization.

## Supporting Information

S1 Dataset Sink at waist. (ZIP)

S2 Dataset Sink at ankle. (ZIP)
